# iPathology cockpit diagnostic station: validation according to College of American Pathologists Pathology and Laboratory Quality Center recommendation at the Hospital Trust and University of Verona

**DOI:** 10.1186/1746-1596-9-S1-S12

**Published:** 2014-12-19

**Authors:** Matteo Brunelli, Serena Beccari, Romano Colombari, Stefano Gobbo, Luca Giobelli, Andrea Pellegrini, Marco Chilosi, Maria Lunardi, Guido Martignoni, Aldo Scarpa, Albino Eccher

**Affiliations:** 1 Department of Pathology and Diagnostics and Hospital Trust, University of Verona, Italy; 2 Fra Castoro Hospital, San Bonifacio Verona ULSS20, Italy; 3 IT Department CED, AOUI, Verona, Italy; 4 ARC-net, Verona, Italy

## Abstract

**Background:**

Validation of digital whole slide images is crucial to ensure that diagnostic performance is at least equivalent to that of glass slides and light microscopy. The College of American Pathologists Pathology and Laboratory Quality Center recently developed recommendations for internal digital pathology system validation. Following these guidelines we sought to validate the performance of a digital approach for routine diagnosis by using an iPad and digital control widescreen-assisted workstation through a pilot study.

**Methods:**

From January 2014, 61 histopathological slides were scanned by ScanScope Digital Slides Scanner (Aperio, Vista, CA). Two independent pathologists performed diagnosis on virtual slides in front of a widescreen by using two computer devices (ImageScope viewing software) located to different Health Institutions (AOUI Verona) connected by local network and a remote image server using an iPad tablet (Aperio, Vista, CA), after uploading the Citrix receiver for iPad. Quality indicators related to image characters and work-flow of the e-health cockpit enterprise system were scored based on subjective (high vs poor) perception. The images were re-evaluated two weeks apart.

**Results:**

The whole glass slides encountered 10 liver: hepatocarcinoma, 10 renal carcinoma, 10 gastric carcinoma and 10 prostate biopsies: adenocarcinoma, 5 excisional skin biopsies: melanoma, 5 lymph-nodes: lymphoma. 6 immuno- and 5 special stains were available for intra- and internet remote viewing. Scan times averaged two minutes and 54 seconds per slide (standard deviation 2 minutes 34 seconds). Megabytes ranged from 256 to 680 (mean 390) per slide storage. Reliance on glass slide, image quality (resolution and color fidelity), slide navigation time, simultaneous viewers in geographically remote locations were considered of high performance score. Side by side comparisons between diagnosis performed on tissue glass slides versus widescreen were excellent showing an almost perfect concordance (0.81, kappa index).

**Conclusions:**

We validated our institutional digital pathology system for routine diagnostic facing with whole slide images in a cockpit enterprise digital system or iPad tablet. Computer widescreens are better for diagnosing scanned glass slide that iPad. For urgent requests, iPad may be used. Legal aspects have to be soon faced with to permit the clinical use of this technology in a manner that does not compromise patient care.

## Background

Pathology labs are coming under pressure to increase their efficiency in diagnostic process timing and improving quality. By digitizing glass slides that pathologists view through a microscope, digital pathology systems offer integrated solutions that help to enhance the efficiency and productivity of pathology departments. Overall, digital systems have been developed with different characteristics and sophisticated yet easy-to-use devices have been designed around the needs of pathologists offering high resolution images. Image quality is the final result of interaction between different components, such as slide scanners, network connections, software systems and monitor workstation, each one with a potential impact on slide image interpretation [1,2]. Respect to the many possible benefits, like any other medical device intended for clinical purposes, there is the need to follow specific regulatory requirements not to compromise patient care [3]. Validation of applications for digital whole slide images for routine diagnostic use is a critical step to ensure that accuracy is at least equivalent to the one obtained viewing glass slide through a light microscopy. For this purpose an expert non vendor panel convened by the College of American Pathologists Pathology and Laboratory Quality Center recently developed practice guidelines for internal digital pathology system validation, recommending a study of intra-observer diagnostic concordance between digitized and glass slides in at least 60 routine cases for each application, viewed at least 2 weeks apart [4]. Since these guidelines are applicable to any whole slide imaging system, we sought to validate the performance of a digital approach for routine diagnosis with images available for intra- and internet remote viewing by using an iPad tablet compared to a widescreen-assisted digital workstation through a pilot study.

## Methods

### Tissue samples

From January 2014, sixty-one histopathological slides from routine diagnostic cases were scanned by ScanScope Digital Slides Scanner (Aperio, Vista, CA) and evaluated at least after 2 week apart from the diagnosis previously performed on glass slides. The time taken for scanning procedures was recorded.

### Cockpit digital station

Two independent pathologists performed diagnosis on virtual slides in front of a widescreen by using two computer devices (ImageScope viewing software) located to different Health Institutions (Verona) connected by local network and a remote image server using an iPad tablet (Aperio, Vista, CA), after uploading the Citrix receiver for iPad. Confidential access to digital systems was set-up for each pathologist.

### Quality indicators and evaluation

Quality indicators related to image characters and to work-flow of the e-health cockpit enterprise system were scored based on subjective (high versus poor) perception. Variables taken into consideration and scored were reliance on glass slide, image resolution and color fidelity, slide navigation time and vision simultaneity in geographically remote locations. Side by side concordance between diagnosis performed on tissue glass slides versus computer widescreen and iPad tablet monitor was also evaluated.

## Results

**Figure 1:**
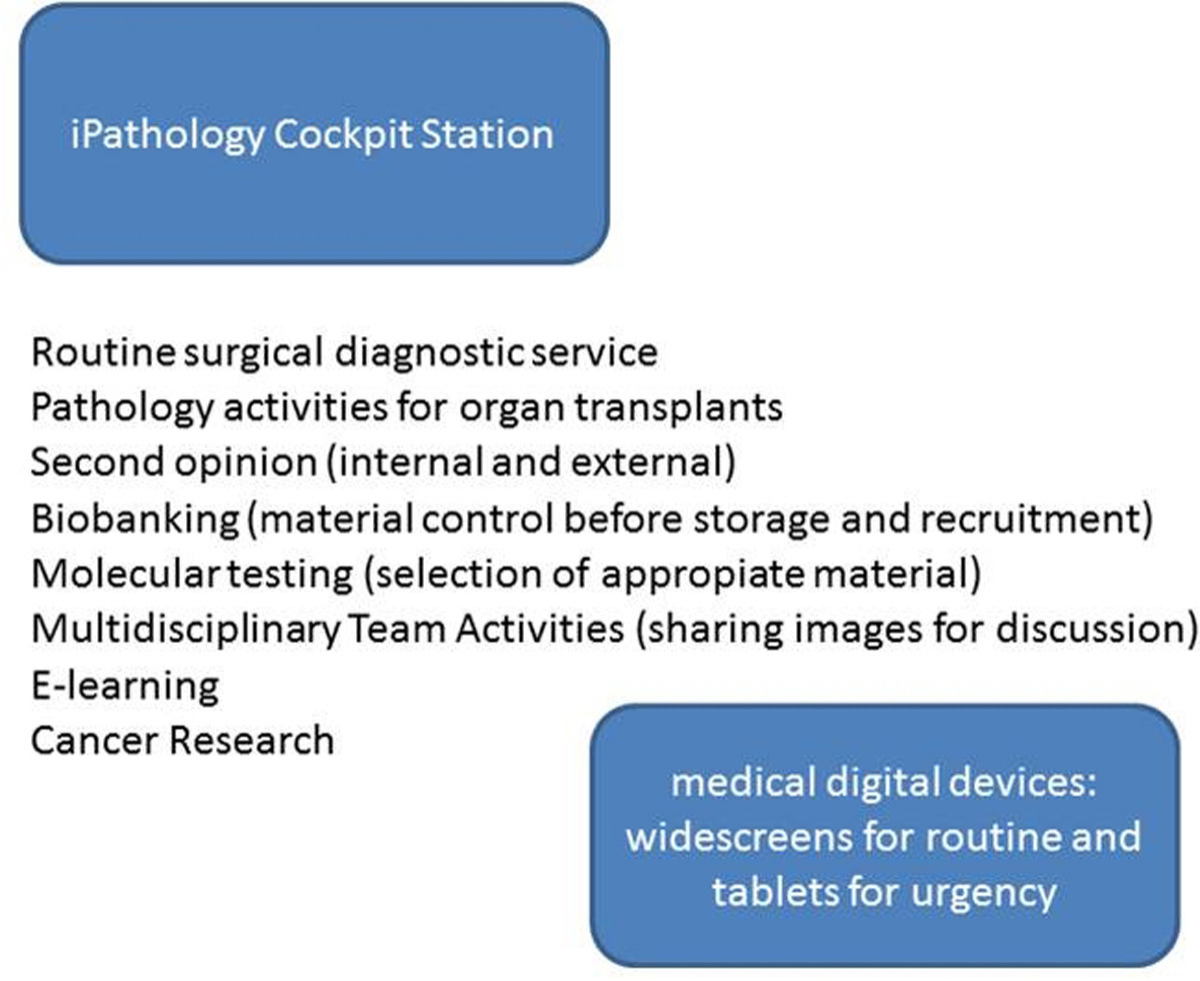
**iPathology activities**.

Routine cases selected for whole slides image analysis (Table 1 and Figure 1) consisted of 10 liver with hepatocarcinoma, 10 renal carcinoma, 10 gastric carcinoma and 10 prostate biopsies with adenocarcinoma, 5 excisional skin biopsies with melanoma and 5 lymph-nodal specimens with lymphoma, all in standard hematoxylin and eosin stain.

**Table 1:** Detailed type of specimens evaluated and other stainings among 61 digitalized cases.

Haematoxylin and eosin		
liver	hepatocarcinoma	10
kidney	renal cell carcinoma	10
gastric	carcinoma	10
prostate	adenocarcinoma	10
lymph-nodes	lymphoma	5
skin biopsy	melanoma	5

**Immunohistochemistry**		

CD20	B lymphoma +	1
CD3	T lymphocites in node+	1
CK8-18	carcinoma +	1
vimentin	sarcoma	1
S100	liposarcoma +	1
Ki67%	high grade B lymphoma	1

**Special stainings**		

Alcian-PAS	bowel	1
Von Kossa	cyst	1
Perls	liver	1
Fontana Masson	cutis	1
Masson trichrome	dermis	1

6 immuno- and 5 special stains were available for intra- and internet remote viewing.

Scan times averaged two minutes and 54 seconds per slide (standard deviation 2 minutes 34 seconds). All quality indicators were considered of high performance score. Megabytes ranged from 256 to 680 (mean 390) per slide storage. Side by side comparisons between tissue glass slides versus widescreen diagnostic performance were excellent, showing an almost perfect concordance (0.81 kappa statistic). Use of iPad was less confident for routine work-flow, due to small size monitor, but suitable for urgency.

## Discussion

Pathology labs are under continued pressure to increase efficiency. Advances in tailored medicine led to a data amount that helps clinicians to target the right therapy at the right patient at the right time. This ever-increasing knowledge about the biology of tumor growth is leading to the demand of more extensive analysis on biopsy tissue and to increasing specialization among pathologists, requiring collaborative diagnostic processes, making it even more difficult for institute managers to balance departmental workloads against available resources. Nevertheless, decreasing the 'time-to-result' in order to speed diagnosis is essential to improving patient outcomes by offering timely and optimal treatment. Pathology remains a largely qualitative process. Extensive market research shows that, even after an extensive assessment, a pathologist's diagnosis can be ambiguous in complex cases. Satisfying these currently unmet needs in modern pathology is the focus of digital pathology systems, offering the possibility of safe diagnostic performance on digital whole slide images [5-7]. Despite the increasing availability of solutions offered by the manufacturers, for the effective translation of new digital pathology concepts into practice there is the need of validation studies joining leading academic, clinical and industrial partners [8]. We validated the digital process when diagnosing scanned slides, facing with whole slide images in a cockpit enterprise (Department of Pathology and Diagnostics, University and Hospital Trust of Verona) digital system compared to an iPad tablet. Guidelines for internal digital system validation by the College of American Pathologists Pathology and Laboratory Quality Center state to re-validate the whole system if one of the component that may affect the slide interpretation is modified. The use of mobile devices in clinical settings has been recently approved by Food and Drug Administration for radiologic images [9]. In literature previous efforts of diagnostic digital pathology with the use of mobile devices like iPad have reported feasibility with good image quality and resolution resulting in acceptable diagnostic accuracy [10]. In our experience computer widescreens are best for facing and finally diagnosing scanned glass slide, in respect to iPad, that may be used anyway for urgent requests. Legal aspects have to be soon faced with to permit the clinical use of this technology in a manner that does not compromise patient care.

## Conclusions

The Hospital Trust and University of Verona set up a digital enterprise diagnostic station (cockpit-cabined) to validate the digital diagnostic processes of pathologists according to the College of American Pathology and Quality Laboratory Center recommendations.

## Competing interests

The authors declare that they have no competing interests.

All Authors declared non-financial competing interests.

## Authors' contributions

1) have made substantial contributions to conception and design, or acquisition of data, or analysis and interpretation of data (MB, AS, RC, AE); 2) have been involved in drafting the manuscript or revising it critically for important intellectual content (SB, ML, MC, GM); 3) have given final approval of the version to be published; and 4) agree to be accountable for all aspects of the work in ensuring that questions related to the accuracy or integrity of any part of the work are appropriately investigated and resolved (LG, AP, SG).
